# Assessing varicella vaccine effectiveness and its influencing factors using health insurance claims data, Germany, 2006 to 2015

**DOI:** 10.2807/1560-7917.ES.2017.22.17.30521

**Published:** 2017-04-27

**Authors:** Thorsten Rieck, Marcel Feig, Matthias an der Heiden, Anette Siedler, Ole Wichmann

**Affiliations:** 1 Robert Koch Institute, Department for Infectious Disease Epidemiology, Berlin, Germany; 2 Charité - University Medicine Berlin, Berlin, Germany

**Keywords:** vaccine effectiveness, varicella virus infection (chickenpox), vaccination coverage, herd immunity, administrative data, immunization information system

## Abstract

In Germany, routine childhood varicella vaccination was implemented in 2004 with two doses recommended since 2009. We used an immunisation information system based on countrywide health insurance claims data to analyse vaccine effectiveness (VE) and factors influencing VE. We applied proportional hazard models to estimate VE under various conditions and compared the risk of acquiring varicella among unvaccinated children in regions with high vs low vaccination coverage (VC). Among 1.4 million children we identified 29,404 varicella cases over a maximum follow-up of 8 years post-vaccination. One-dose VE was 81.9% (95% confidence interval (CI): 81.4–82.5), two-dose VE 94.4% (95% CI: 94.2–94.6). With dose one given 1–27 days after measles-containing vaccine (MCV), one-dose VE was 32.2% (95% CI: 10.4–48.6), two-dose VE 92.8% (95% CI: 84.8–96.6). VE was not associated with age at vaccination (11–14 vs ≥ 15 months), time since vaccination, or vaccine type. Unvaccinated children had a twofold higher risk of acquiring varicella in low VC regions. Our system generated valuable data, showing that two-dose varicella vaccination provides good protection for at least 8 years. Unvaccinated children benefit from herd effects. When the first varicella vaccine dose is given shortly after MCV, a second dose is essential.

## Introduction

Immunisation Information Systems (IIS) are defined by the Centers for Disease Control and Prevention as confidential, population-based, computerised databases that record all immunisation doses administered by participating providers to persons residing within a given geopolitical area [1]. At the point of clinical care, IIS may support vaccination providers in decision-making towards appropriate individual vaccinations. At the population level, IIS provide aggregate data on vaccinations for use in surveillance and programme operations, and in guiding public health action with the goals of improving vaccination rates and reducing vaccine-preventable disease. In 2004, Germany started to implement a nationwide IIS for the monitoring of vaccination coverage (VC) and selected vaccine-preventable diseases based on health insurance claims data. The German IIS covers the statutory health-insured population (ca 85% of the total population in Germany) and has proved to be a reliable source of VC data [2-5]. Moreover, the data were used to estimate the incidence of selected vaccine-preventable diseases such as measles, mumps and herpes zoster in Germany [6-8]. Varicella is primarily clinically diagnosed [9], thus the German IIS seems suitable for the identification of varicella cases in the population.

Germany is one of the few countries worldwide that has introduced routine childhood varicella vaccination [10]. Since 2004, single-dose varicella vaccination has been recommended for all children aged 11–14 months. Two single-compound varicella vaccines (VAR; Varivax, Sanofi Pasteur MSD; Varilrix, GlaxoSmithKline) were initially available. In 2006, a combined measles-mumps-rubella-(MMR)-varicella vaccine (MMRV; Priorix-Tetra, GlaxoSmithKline) was licensed with a two-dose schedule. A universal two-dose schedule has been recommended since 2009 targeting children with the second dose at age 15–23 months. Since 2011, the first immunisation has been given preferably as two separate injections of VAR and MMR due to higher rates of febrile seizures following immunisation with MMRV [11]. Catch-up vaccinations are recommended until 17 years of age.

The impact of routine varicella vaccination was initially monitored in a countrywide physician-based sentinel system. Sentinel data indicated a continuous overall 84% decrease of varicella cases per sentinel site between 2005 and 2012, most dominantly among 1–4 year-olds [12]. Based on data from the IIS, VC in 24-month-old children increased nationwide in subsequent birth cohorts 2004–2009 from 43% to 87% (at least one dose) and from 1% to 64% (two doses) [3], whereas in the federal state of Saxony, varicella VC increased from 33% to 76% (at least one dose) and from < 1% to 24% (two doses). Within each birth cohort, the lowest VC was identified in the federal state of Saxony.

Several post-marketing studies on varicella vaccine effectiveness (VE) have been published [13-22]. However, of these, only few studies assessed the effectiveness of two doses [13-16]. In addition, little is known about the duration of vaccine-induced protection and the optimal age for vaccination [17-19]. Finally, there is little evidence on the minimum time interval between the first and second varicella vaccine dose as well as between varicella and measles-virus containing vaccines (MCV) [18,20].

We used data from the German IIS with the objectives to estimate dose-specific VE against all varicella, varicella-associated complications and varicella without complications, and to investigate factors that might influence VE, such as age at vaccination, time interval between varicella and MCV doses, type of vaccine, and time since vaccination (TSV). Furthermore, we aimed to quantify the degree of herd protection that is conferred in regions with high vs low VC.

## Methods

### Dataflow and database

Data were generated and collected within the German IIS, also called the ’Associations of Statutory Health Insurance Physicians (ASHIPs) vaccination monitoring project’. The system has been described in detail previously [3]. In brief, ASHIPs regularly receive insurance refund claims from all ASHIP-associated physicians for outpatient medical services provided to those covered by statutory health insurance. These claims data include all recommended vaccinations and diagnosed diseases. The latter have to be documented in order to justify medical services. Approximately 85% of the population in Germany is covered by statutory health insurance. The remainder are mainly privately insured. The administrative regions of most ASHIPs are organised by federal state. Data relevant for the project are extracted from the ASHIPs’ databases and anonymised. Data are quarterly transferred to the Robert Koch Institute (RKI, German national public health institute), and imported into a central database. Since 2006, the database contains patient information, data on vaccinations and diagnoses of selected vaccine-preventable diseases, and since 2008, dates of individuals’ physician consultations (Table 1).

**Table 1:** Database content relevant to varicella disease and vaccination in the German Associations of Statutory Health Insurance Physicians vaccination monitoring project

**Patient information**	
Anonymised unique identifier Month/year of birth Sex County of residence	
**Vaccination information**	
Claim codes of all recommended vaccinations (antigen or antigen combination specific) Date of vaccination	
**Diagnosis information**	
Varicella-specific ICD-10 codes [38]	B01. Varicella [chickenpox] B01.0 Varicella meningitis B01.1 Varicella encephalitis B01.2 Varicella pneumonia B01.8 Varicella with other complications B01.9 Varicella without complication
Diagnosis type	Current state Previous state Unknown Not provided
Diagnosis reliability	Suspected Confirmed Recovered Excluded
Quarter and year of diagnosis	
**Physician contact information**	
Physician’s ASHIP Date of patients’ first contact per quarter and medical specialisation	

ICD-10: International Classification of Diseases, 10th revision [38]; ASHIP: Associations of Statutory Health Insurance Physicians.

### Data protection

The Federal Commissioner for Data Protection and Freedom of Information in Germany has approved the ASHIP vaccination monitoring project.

### Sampling and data preparation

The unique patient identifier in the anonymised data is generated differently between ASHIPs. Therefore, medical services received by a single patient can only be assigned to a unique patient identifier in the data anonymised by a single ASHIP but not by different ASHIPs. As a consequence, we would identify an individual receiving the first varicella vaccine dose in one ASHIP region and the second dose in another region (e.g. due to moving into another federal state) as two individuals in the central database, both with incomplete vaccination series. For this reason, we selected individuals according to inclusion criteria as described previously [2,3]: Any individual (i) born between January 2006 and October 2013, (ii) receiving any vaccination (i.e. not necessarily varicella) soon after birth at 0–4 months of age, (iii) with contact with a physician within the second half of 2015, (iv) residing at the time points of (ii) and (iii) in the region of the ASHIP that transferred the data, and (v) born in an ASHIP region where diagnosis information was available and specific vaccination claim codes for varicella vaccines had been introduced since birth. Within this sampling period, i.e. from birth to the second half of 2015, the actual analysis time in the follow-up period went from the quarter in which the child turned 11 months until June 2015 at maximum. We assumed included children presented at physicians exclusively within their associated ASHIP region during the follow-up period because both in the beginning and in the end of the period, physician contacts were documented within their resident ASHIP region.

Data from 12 of 17 ASHIP regions were analysed, starting from either birth cohort 2006 (ASHIPs Brandenburg, Hamburg, Mecklenburg-West Pomerania, Lower Saxony, Saarland, Saxony-Anhalt, Schleswig-Holstein, Thuringia) or later birth cohorts due to late introduction of MMRV-specific claim codes, i.e. birth cohort 2007 (ASHIP Saxony) or 2008 (ASHIPs Baden-Württemberg, North Rhine, Berlin). Data from the remaining ASHIPs were either missing for several years, did not contain the variable ‘diagnosis type’, or were incomplete regarding physician contacts.

We applied a four-step algorithm to only select confirmed and incident (diagnosis type: current state) varicella diagnoses and to limit these to the earliest and to the most severe varicella diagnosis for each selected patient as described previously [6,7]. Briefly, step 1 excluded incompatible or implausible coding combinations for varicella diagnosis reliability; step 2 excluded observations with diagnosis reliability other than confirmed (i.e. suspected, excluded, recovered); step 3 excluded observations with diagnosis type other than incident (i.e. previous state, unknown, not provided); step 4 limited the data selection to the earliest ICD-10 code per patient while, in addition, keeping the information about the most severe ICD-10 code (within up to one quarter following the initial diagnosis) using the following ranking (in descending order of severity): varicella with encephalitis, meningitis, pneumonia, other complications, no complications, no further details, with the latter equalling ‘no complications’.

The date of diagnosis was quarter-specific. Therefore, the unit of analysis time used in our models was one quarter of a year. Individual analysis time in the models started with the quarter in which the child turned 11 months of age (i.e. the ’entry’ in the time-series analysis), and lasted until the last quarter of the follow-up period (i.e. the ‘exit’). We reduced the vaccination date from day-specific to quarter-specific for calculations of analysis time. Children with varicella vaccinations and/or a varicella diagnosis before the entry quarter were excluded from VE analysis. Due to the granularity of the date of diagnosis, we could reliably identify breakthrough infections, defined as varicella infection being diagnosed ≥ 42 days post vaccination, only when the vaccine was received at least three quarters preceding the diagnosis. We therefore excluded the first two quarters of analysis time of each vaccination status of a patient in the time-series models. Hence, this excluded patients with half a year or less of analysis time. We also excluded children where sex was not recorded and those with presumably erroneous documented VAR plus MMRV or MMRV plus MMR vaccinations on the same day.

### Data analysis

Individual histories of first and second varicella vaccination and varicella diagnosis were set up for time-series analysis in Stata 13 (StataCorp, US). We used Cox-like piecewise proportional hazard models allowing for the analysis of potentially varying hazard ratios over TSV. We stratified the observations at an individual level into the period of > 0.5–1.0 years TSV and seven annual periods from > 1.0–2.0 to > 7.0–8.0 years TSV to analyse VE by TSV. For all other analyses of VE, we did not stratify the observation periods but performed the analysis over the whole TSV beginning at > 0.5 years TSV. The individual analysis time either ended due to censoring or failure, respectively, or stopped with each change of vaccination status while restarting at zero with assigning the patient’s new vaccination status.

We verified the proportional hazard assumption – a prerequisite for modelling Cox regression for time-series data – for each covariate and globally at the stratum level using formal significance tests and graphical evaluation of unscaled and scaled Schoenfeld residuals. Additionally, we performed graphical assessment of proportional hazards using log-log survival curves.

In the Cox regression model we used vaccination status and TSV as the categorical predictor variables. We stratified by sex, year of birth, and ASHIP to ensure comparing children of similar age and region. Strata were weighted using probability weights generated from sex-, birth cohort-, and ASHIP region-specific live-births and sample size (German Federal Statistical Office; State Office for Information and Technology North Rhine-Westphalia).

We calculated VE as ‘(1-hazard ratio) x 100’, and modelled incremental VE as the additional effectiveness provided by the second dose compared with a single dose ([VE2-VE1] / [100-VE1] x 100). We calculated VE in the whole sample but also by exclusion of children with incompliant spacing of vaccinations, i.e. varicella vaccinations given 1–27 days after MCV or subsequent varicella vaccine doses administered within 1–27 days. In addition, we built models which were either extended by the inclusion of variables for (i) type of vaccine (VAR, MMRV), (ii) age at first varicella vaccination (11–14 months; > 15 months of age), (iii) time to varicella vaccination following MCV (same day as MCV or > 27 days after MCV; incompliant spacing) excluding patients with incompliant spacing between varicella vaccinations, or (iv) time between first and second varicella vaccination (incompliant spacing; 28–365 days; > 1–3 years; > 3 years) excluding patients with incompliant spacing to MCV. We defined one group of outcome (no complications) as failure and censored the patient in presence of the remaining outcome group (complications) and vice versa to estimate VE for the prevention of varicella-associated complications vs no complications. Using the Wald test, we tested for significant differences of coefficients and their interactions and applied a Bonferroni correction to the p values when multiple testing was performed.

The cumulative baseline hazards in the time-series analyses are the stratum-specific cumulative hazards in unvaccinated children. We used these to estimate morbidity among unvaccinated children stratified by sex, birth cohort and ASHIP. We used linear regression to summarise these cumulative hazards for both Saxony, an area with low VC, and the remaining ASHIP regions. The ratio of these mean cumulative hazards was used to compare the different degree of protection in regions with different VC. The risk R(t) of acquiring varicella up to time t can be derived from the cumulative hazard H(t) by applying the formula $R\left(t\right)=1-e{\;}^{-H\;\left(t\right)}$. In addition, we calculated attack rates from the observational data using probability weights (Pearson test statistics of the survey procedures in Stata) for Saxony and outside Saxony and compared them with each other in a rate ratio. We set the significance level to 0.05.

We calculated longitudinal VC both in Saxony and the remaining ASHIP regions as described previously by counting age-specific doses at an individual level by ASHIP/year of birth/sex and subsequent aggregation to VC by age within and outside Saxony [2,3].

## Results

Between January 2006 and October 2013, a total of 5,294,301 live births were registered in Germany, of which 2,790,220 children (53%) were born in the investigated ASHIP regions and years of birth. Among those, 1,449,411 children (52%) were available for VE analysis (range over regions and years of birth: 35–69%). Their characteristics are given in Table 2.

**Table 2:** Data characteristics of individuals analysed in the time-series models for varicella vaccine effectiveness estimates, Germany, 2006–2015

**Characteristics**	**Measure**
Number of subjects (%)	1,449,411 (100)
Number of females (%)	704,036 (48.6)
Number of varicella cases (%)	29,404 (2.0)
Mean years of age at diagnosis	3.6
**Number of cases with complications (% among cases)**	1,213 (4.13)
Encephalitis (% among cases)	33 (0.11)
Meningitis (% among cases)	129 (0.44)
Pneumonia (% among cases)	9 (0.03)
Other (% among cases)	1,042 (3.54)
**Mean years of individual analysis time (total person‑years)**	3.0 (4,332,641)
**Number of individuals receiving varicella vaccination (%)**	
No vaccination	92,712 (6.4)
1st dose	1,298,697 (89.6)
2nd dose	1,090,969 (75.3)
**Number of administered vaccine type (%)**	
1st VAR	490,002 (33.8)
1st MMRV	808,695 (55.8)
2nd VAR	87,504 (6.0)
2nd MMRV	1,003,465 (69.2)
**Mean months of age at vaccination**	
1st dose	15
2nd dose	22
**Number of individuals receiving 1st vaccination by age (% among 1st doses)**	
11–14 months	1,030,331 (79.3)
≥ 15 months	268,366 (20.7)
**Number of subjects with varicella vaccination after MCV (%)**	
At least one dose 1–27 days	5,434 (0.4)
All doses same day or > 27 days	1,293,263 (89.2)
**Number of 2nd vaccinations by distance to 1st dose (% among 2nd doses)**	
1–27 days	2,862 (0.3)
28–365 days	919,711 (84.3)
> 1 year–3 years	148,198 (13.6)
> 3 years	20,198 (1.9)

MCV: measles containing vaccine; MMRV: measles-mumps-rubella-varicella vaccine; VAR: single-compound varicella vaccine.

### Overall vaccine effectiveness

Over the total observation period and after exclusion of children with incompliant spacing between vaccine doses, VE for one dose (VE1) was 81.9% (95% confidence interval (CI): 81.4–82.5) and significantly lower (p < 0.0001) than VE2 with 94.4% (95% CI: 94.2–94.6) (Table 3). The incremental VE of adding a second dose to the first dose was 68.9% (95% CI: 67.5–70.1). Stratified by sex, the VE1 difference was less than 2 percentage points and slightly higher in females as compared with males (82.8% vs 81.1%, p = 0.0015); for VE2, the difference was even smaller (94.6% vs 94.2%, p = 0.0072). The inclusion of children with incompliant spacing had nearly no influence on VE (Table 3).

**Table 3:** Varicella vaccine effectiveness from > 0.5 to 8.0 years since vaccination based on estimates from time-series analysis, Germany, 2006–2015 (n = 1,449,411)

Overall (excluding patients receiving varicella vaccinations 1–27 days after MCV or 1st and 2nd dose varicella 1–27 days apart)			VE1 (95% CI)	VE2 (95% CI)	
			81.9 (81.4–82.5)	94.4 (94.2–94.6)	
Overall			81.8 (81.2–82.4)	94.4 (94.2–94.6)	
Age at 1st vaccination^a^					
	11–14 months		82.1 (81.4–82.8)	NA	
	≥ 15 months		81.5 (80.6–82.3)		
Varicella vaccination after MCV (excluding patients receiving 1st and 2nd dose varicella 1–27 days apart)^b^					
				2nd dose 1–27 days	2nd dose same day or > 27 days
	1st dose 1–27 days		32.2 (10.4–48.6)	No meaningful estimate (n = 26; 1 varicella case)	92.8 (84.8–96.6)
	1st dose same day or > 27 days		80.9 (80.2–81.5)	95.3 (66.6–99.3)	94.1 (93.9–94.3)
	
Time interval 1st to 2nd dose (excluding patients receiving varicella vaccinations 1–27 days after MCV)^c^					
	1–27 days		NA	87.3 (61.3–95.8)	NA
	28–365 days			94.4 (94.2–94.6)	
	> 1–3 years			94.8 (94.4–95.2)	
	> 3 years			95.0 (93.6–96.1)	
Vaccine type^d^					
				2nd dose VAR	2nd dose MMRV
	1st dose VAR		82.0 (81.0–82.9)	95.0 (94.3–95.5)	94.3 (93.9–94.8)
	1st dose MMRV		81.7 (81.0–82.4)	94.4 (93.4–95.2)	94.4 (94.2–94.6)
Prevention of uncomplicated/complicated cases^e,f^					
	No complication	65.3 (64.2–66.4)		89.3 (89.0–89.7)	NA
	All complications	98.2 (98.0–98.5)		99.5 (99.4–99.5)	

CI: confidence interval; MCV: measles containing vaccine; MMRV: measles-mumps-rubella-varicella combination vaccine; NA: not applicable; VAR: single-compound varicella vaccine; VE: vaccine effectiveness; VE1: vaccine effectiveness for one dose; VE2: vaccine effectiveness for two doses.

^a^ VE1 difference not significant.

^b^ Within VE1, VE is significantly different (p < 0.0001); within VE2 and where applicable, no combination with VE from both doses administered 0 / > 27 days apart significantly different.

^c^ No combination with VE at 28–365 days significantly different.

^d^ Within VE1 and VE2, no combination significantly different.

^e^ In contrast to the outcome ‘varicella’ in the majority of models, here we defined ‘varicella without complications’ as failure and censored the patient in presence of ‘varicella with associated complications’ and vice versa to estimate VE.

^f^ Within VE1 and VE2 and between VE1 and VE2 difference significant (all p < 0.0001).

All given VE estimates are significant.

### Vaccine effectiveness by time since vaccination and age

While VE1 increased over time from 79.4% (95% CI: 78.2–80.5) at > 0.5–1.0 year TSV to 88.0% (95% CI: 76.6–93.8) at > 7.0–8.0 years TSV, VE2 remained stable at > 92.0% (Table 4).

**Table 4:** Varicella vaccine effectiveness by time since vaccination and vaccine-type estimated from time-series analysis using administrative data and effective sample size, Germany, 2006–2015

Time since vaccination (years)	VE1 (95% CI)	VE1 (95% CI) VAR	VE1 (95% CI) MMRV	VE2 (95% CI)	Effective sample size			
					**n^a^**	**n_0dose_**	**n_1dose_**	**n_2dose_**
> 0.5–1.0	79.4 (78.2–80.5)	80.6 (78.8–82.3)	78.0 (76.4–79.5)	93.1 (92.7–93.5)	1,449,411	275,814	527,514	1,090,969
> 1.0–2.0	82.2 (81.2–83.1)^b^	84.0 (82.5–85.3)^b^	81.0 (79.7–82.2) ^b^	94.2 (93.9–94.5) ^b^	1,259,119	176,424	264,220	972,827
> 2.0–3.0	82.7 (81.6–83.8) ^b^	82.1 (80.0–84.0)	83.5 (82.1–84.7) ^b^	95.3 (95.0–95.5) ^b^	956,643	101,550	127,393	756,329
> 3.0–4.0	82.8 (81.3–84.2)^b^	81.6 (78.4–84.4)	83.7 (82.0–85.1) ^b^	94.8 (94.4–95.2) ^b^	708,054	65,970	79,938	566,342
> 4.0–5.0	82.4 (80.1–84.4)	79.1 (73.5–83.5)	83.5 (81.0–85.6) ^b^	94.7 (94.2–95.2) ^b^	467,703	43,092	48,090	376,531
> 5.0–6.0	84.1 (80.6–87.0)	81.7 (72.7–87.7)	85.3 (81.5–88.3) ^b^	95.0 (94.2–95.7) ^b^	265,351	26,302	25,218	213,831
> 6.0–7.0	85.7 (79.9–89.9)	78.1 (60.9–87.8)	87.4 (80.7–91.8) ^b^	93.3 (91.7–94.6)	114,503	13,249	12,172	89,082
> 7.0–8.0	88.0 (76.6–93.8)	78.4 (47.7–91.1)	92.0 (78.3–97.1) ^b^	92.4 (88.3–95.0)	40,806	6,038	5,050	29,718

CI: confidence interval; MMRV: measles-mumps-rubella-varicella vaccine; VAR: single-compound varicella vaccine; VE1: vaccine effectiveness for one dose; VE2: vaccine effectiveness for two doses.

^a^ Total sample may be smaller than the sum of vaccination status specific sample sizes as a single patient may have several vaccination statuses within one analysis period.

^b^ Within VE1 or VE2, respectively, significantly different to VE > 0.5–1.0 years since vaccination.

Within the first year since vaccination, both VE1 and VE2 were slightly but significantly lower than in the following 3–5 time intervals. When stratifying by vaccine type, VE1 from VAR stayed in the same magnitude over time (80.6% (95% CI: 78.8–82.3) at > 0.5–1.0 year TSV; 78.4% (95% CI: 47.7–91.1) at > 7.0–8.0 years TSV) whereas VE1 from MMRV increased from 78.0% (95% CI: 76.4–79.5) to 92.0% (95% CI: 78.3–97.1). VE1 did not differ by age at first vaccination (82.1%; 95% CI: 81.4–82.8 in age group 11–14 months and 81.5%; 95% CI: 80.6–82.3 at *≥* 15 months; Table 3).

### Effect of time between subsequent live attenuated vaccine doses

A single varicella vaccination given 1–27 days after MCV conferred significantly lower protection (VE1 = 32.2%, 95% CI: 10.4–48.6) than a single dose given simultaneously or > 27 days after MCV (80.9%, 95% CI: 80.2–81.5) (Table 3). VE2 was not reduced when only one of the two doses was administered 1–27 days after MCV.

Two varicella vaccinations administered in an interval of 28–365 days gave statistically similar VE2 as vaccinations given 1–27 days, > 1–3 years or > 3 years apart. The VE2 estimate for varicella vaccinations 1–27 days apart was based on only 305 patients, was ca 8 percentage points lower than with any other time interval and had a wide 95% CI.

### Vaccine effectiveness by vaccine type

We found similar VE1 for VAR and MMRV (82.0% (95% CI: 81.0–82.9) vs 81.7% (95% CI: 81.0–82.4), respectively) (Table 3). VE2 for all combinations of VAR and MMRV as first or second dose were also similar and ranged between 94.3% (95% CI: 93.9–94.8) and 95.0% (95% CI: 94.3–95.5).

### Protection against complicated vs non-complicated varicella

VE against varicella-associated complications (VE1 = 98.2%, 95% CI: 98.0–98.5; VE2 = 99.5%, 95% CI: 99.4–99.5) was significantly higher than against non-complicated varicella (VE1 = 65.3%, 95% CI: 64.2–66.4; VE2 = 89.3%, 95% CI: 89.0–89.7) with two doses being significantly more effective than a single dose.

### Risk of acquiring varicella among unvaccinated children

**Figure 1:**
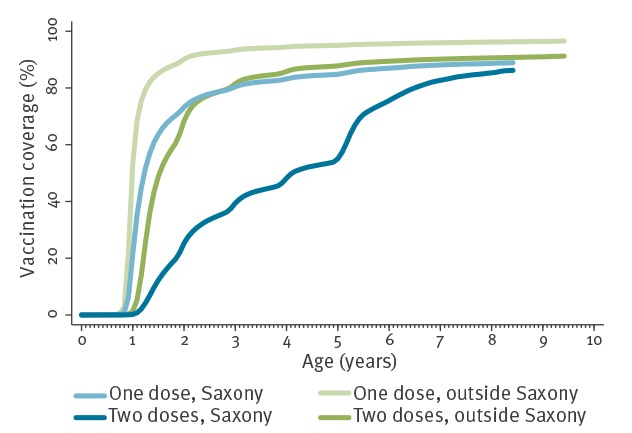
Cumulative coverage by age in the federal state of Saxony (n = 179,162) and other regions of Germany (n = 1,760,220) for one and two varicella vaccinations for all ASHIPs and birth cohorts selected for time-series analysis to estimate vaccine effectiveness, 2006–2015

VC in Saxony was lower than in other ASHIP regions over the whole age-range covered in the sample; e.g. at 24 months of age VC1 was 73.2% and VC2 was 25.3% in Saxony vs 90.1% and 68.3% outside Saxony (Figure 1).

ASHIP: Association of Statutory Health Insurance Physicians.

**Figure 2:**
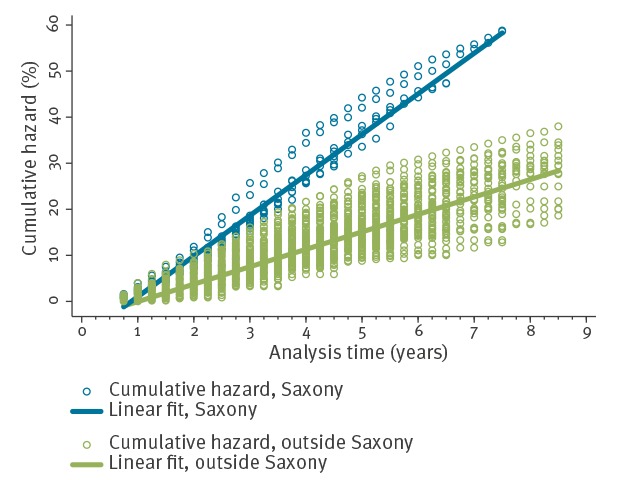
Cumulative hazard in ASHIP/year of birth/sex strata by analysis time in time-series analysis and linear fit among unvaccinated children in the federal state of Saxony (n = 52,441) and other regions of Germany (n = 223,373), 2006–2015

The attack rate of varicella in unvaccinated children was 12.6% (95% CI: 12.3–12.9) in Saxony vs 5.8% (95% CI: 5.7–5.9) in other regions translating into a rate ratio of 2.2 (95% CI: 2.1–2.2; p < 0.0001). The cumulative hazard and the risk of acquiring varicella among unvaccinated children were around two times higher in Saxony; e.g. after 4 years of analysis time we calculated a ratio of the cumulative hazards of 2.4 and a risk ratio of 2.2 corresponding to a cumulative hazard of 27.4% (95% CI: 27.4–27.5) in Saxony vs 11.3% (95% CI: 11.3–11.3) outside of Saxony, and after 7.5 years of analysis time the ratio of the cumulative hazards was 2.4 and the risk ratio 2.0, corresponding to a cumulative hazard of 58.3% (95% CI: 58.3–58.3) in Saxony vs 24.6% (95% CI: 24.6–24.6) in other regions (Figure 2).

ASHIP: Association of Statutory Health Insurance Physicians.

Analysis time 0–0.5 years was omitted from hazard analysis. At time = 0, children are ca 11 months.

Linear fit (p < 0.001, R-squared = 0.94).

## Discussion

Starting from diagnoses and administered vaccinations as documented in health insurance claims and linked at the individual level, our analysis shows that the German IIS is a potent system for the continuous monitoring not only of VC but also of the effectiveness of vaccination and the impact of vaccination at the population level after widespread use. Our data confirm the additional effect of a second varicella vaccine dose and demonstrate indirect protection of unvaccinated individuals in areas with high VC.

Evidence on the loss of vaccine-induced protection after one dose has been inconclusive in previous studies [17-19]. Our data demonstrate the absence of waning of vaccine-induced protection by one and two doses over at least eight years. However, after the second dose, protection is much higher in each observed time interval after vaccination, with an overall incremental effectiveness of 68.9%. The result for VE1 is in line with data from a case–control study and a time-series approach in Germany where 86.4% and 83.2% were estimated [21,22]. Our findings are also comparable to the results of a recent German study based on the screening method and to international data from case–control studies where VE1 and VE2 were at 80–87% and 97–98%, respectively [13,14,15,23-26]. In addition, a 2016 meta-analysis of literature published between 1995 and 2014 on VE among healthy children reported similar results with a pooled VE1 of 81% (95% CI: 78–84) and a pooled VE2 of 92% (95% CI: 88–95) [27].

We found similar VE1 irrespective of young or older age at vaccination. Previously, evidence for vaccination at young age as a potential risk factor for vaccine failure has been reported inconclusively [28,29]. Our findings support the current national immunisation scheme recommending the first varicella dose at young age (from as early as 11 months) as recommended in the majority of countries that have adopted varicella vaccination [30].

VE1 was strongly reduced when the first varicella vaccination was administered with incompliant spacing to MCV. In contrast, VE2 estimates were not affected when one of the doses (first or second) were given with incompliant spacing to MCV. Due to small sample size, VE2 under the condition of both doses given with incompliant spacing could not be analysed. A higher risk for varicella due to a short spacing between the administration of MCV and a single varicella vaccine dose has been described previously [29]. Generally, a minimal time interval for the administration of live attenuated vaccines is recommended to avoid potential suppressive effects on the immune response. To our knowledge this has only been studied for vaccinations given up to 4 weeks after MMR vaccinations but not for successive varicella vaccinations [29,31,32]. In contrast to our result that two varicella vaccine doses may compensate the reduced VE of one dose given too early after MCV, the VE2 point estimate of the 1–27-day time interval between varicella vaccine doses was lower than for longer intervals. However, the sample size was small and the decrease was statistically non-significant. Still, this might indicate that a short spacing of subsequent varicella vaccinations negatively affects VE. This is of particular importance for accelerated schedules in situations like outbreaks, urgent catch-ups and for rapid immunisations before travelling. Overall, simultaneous administration of MCV and varicella vaccine or a time interval > 27 days between these vaccinations or between subsequent varicella vaccinations seemed to confer optimal protection against varicella. We found no significant difference in VE2 in all investigated time intervals > 27 days up to > 3years between varicella doses, indicating that different national or regional recommendations regarding this interval will lead to similar VE2. This observation and our result that vaccine-induced protection is not waning, support the current recommendation in Germany for a second dose given early in childhood.

We found no statistical difference in VE from single-compound vs combined vaccines, neither for one dose nor in any two-dose combination. Also at the level of point estimates, VE was virtually similar. Although Spackova et al. identified differences in relative risk point estimates for breakthrough infections by type of vaccine, their findings concerning the use of two single-compound vaccines and MMRV were non-significant [16]. Our finding shows that in particular the currently recommended combination of VAR followed by MMRV in Germany confers the same protection as any other combination.

Our results show that a single dose better protects against a more serious course of infection than against mild varicella. A second dose only adds a small additional benefit in this regard. Our results point towards the same direction as the results of previous observations, although the observed endpoints were different (recorded codes of diagnosis in our study vs observed symptoms or number of lesions in other studies) [27].

We found an around twofold higher attack rate and risk of acquiring varicella in unvaccinated children in Saxony vs other ASHIP regions. Saxony has a much lower VC for both first and second dose varicella vaccination than any other ASHIP region in Germany. Similar findings are annually published based on cross-sectional analyses from nationwide school entrance examinations [33]. Having its own state level advisory committee on immunisation, Saxony recommended until the end of 2014 the second varicella dose from five years of age [34]. The lower risk of acquiring varicella in the unprotected population in regions of Germany that have higher varicella VC than Saxony is a strong indication for the presence of herd effects. Varicella herd protection was described previously based on health insurance claims data showing a decline in varicella outpatient visits and hospitalisations among infants and adults not targeted for vaccination in the United States [35].

The basis for our analyses are health insurance claims data primarily generated for the reimbursement of medical services provided by physicians. They have not been created for the purpose of answering epidemiological questions in secondary data analyses. However, reimbursement for vaccinations is directly linked to correct code usage. Hence, validity of vaccination data can be expected to be very high as we have previously shown [3]. Still, several MMRV claim code changes occurred soon after its availability. Wrong usage will have led to misclassification of a second MMRV dose as the first dose in our IIS, which was more likely in the early years of the programme. This explains the increase of VE1 over higher intervals of TSV from MMRV but not VAR. Therefore, VE1 estimated from VAR may be a more accurate representation of VE over TSV. VE1 measured from both VAR and MMRV in the overall analysis, however, is nearly similar to VE1 measured from VAR alone suggesting that the potential misclassification is of minor consequence.

Our IIS covers all individuals in Germany with statutory health insurance. Between 2006 and 2015, an average of 83% among 0–14 year-olds (range between ASHIP regions: 81–89%) were statutory health insured (statistics of statutory health insurees by the German Ministry of Health; population statistics by the Federal Statistics Office). Both statutory and private health insurances fully reimburse recommended vaccinations. The authors of a large population-based cross-sectional study found no difference in the proportions of undervaccinated children when comparing children from parents with statutory and private health insurance [36]. Thus, we assume comparable VC and VE in children not covered by the IIS.

Diagnoses from health insurance claims data have been exploited for measles incidence estimation and showed trends and variation similar to outpatient notification data estimates supporting their usefulness for epidemiological analyses [7]. However, there are no standardised guidelines for coding and updating diagnoses as ‘confirmed’ or ‘suspected’ disease. The physician does not require laboratory confirmation for this classification and may solely rely on clinical symptoms. Since we used only confirmed cases, our sampling approach for cases might have been rather conservative. Nonetheless, physicians may feel more confident in classifying a diagnosis as confirmed in unvaccinated cases. Because patients with mild disease are less likely to present at their physician while the probability for a mild course of the disease is higher for vaccinated cases, a bias might have been introduced in our study population which would result in an overestimation of overall VE but not VE for the prevention of severe varicella. We identified 4.13% of complications among all cases. This is in line with previous reports of 2–6% of cases with complications attending a general practice [37]. However, since health insurance claims data only cover outpatient data and complicated cases are more likely to be hospitalised and less likely to (at least initially) present as outpatient case, these cases are possibly underrepresented in our sample and therefore not included in the analysis. In 2004, a total of 2,316 hospitalised varicella cases were recorded in the statistics of hospital diagnoses followed by a decreasing trend to around 1,000 cases from 2008 until 2014 and an increase to 1,504 cases in 2015 (Federal Statistics Office). The decrease was especially prominent in children below 5 years of age, ranging from 1,139 cases in 2004 to 159 and 207 cases in 2014 and 2015, respectively. Since severity is associated with not being vaccinated, hospitalised cases among unvaccinated children may be disproportionately underrepresented in our sample. This bias may have led to a slight underestimation of our calculated VE.

Our IIS was implemented in 2004 and – after successful validations and extensive piloting – serves as a unique source to monitor and evaluate vaccination recommendations and strategies in Germany. The system provides VC data for the international reporting to the World Health Organization and informs the National Verification Committee for Measles and Rubella Elimination on the elimination progress in Germany, since it currently offers the only nationwide data source to estimate VC in various age groups. In addition, since vaccination claims and disease codes can be linked at an individual level and the IIS captures a large proportion of the total population, it provides the opportunity to assess VE and vaccination programme impact at a population level. When the German Standing Committee on Vaccination initially endorsed the two-dose recommendation for varicella vaccination, it requested an evaluation by 2013. The IIS was one of four surveillance data sources that contributed to this evaluation [20]. There were some remaining questions that we were able to address in the present study, namely the duration of varicella vaccine-induced protection after two doses, the optimal age for the second dose, and potential differences in VE between the available varicella vaccine types.

By demonstrating that we were able to answer important questions related to the national varicella vaccination programme, we conclude that our IIS is an indispensable system not only for the assessment of VC in various age groups and geographical regions in Germany, but also for the monitoring and in-depth evaluation of national vaccination recommendations and strategies.
